# Evaluation of Mast Cell Density in Mucoepidermoid Carcinoma and Pleomorphic Adenoma

**DOI:** 10.30476/dentjods.2022.93712.1730

**Published:** 2023-06-01

**Authors:** Azadeh Jalili, Najmeh Jafari, Seyed Hosein Tabatabaei

**Affiliations:** 1 School of Dentistry, Shahid Sadoughi University of Medical Sciences, Yazd, Iran; 2 Dept. of Oral and Maxillofacial Pathology, School of Dentistry, Shahid Sadoughi University of Medical Sciences, Yazd, Iran; 3 Dept. of Oral and Maxillofacial Pathology, School of Dentistry, Social Determinant of Oral health Research Center, Shahid Sadoughi University of Medical Sciences, Yazd, Iran

**Keywords:** Mast cells, Carcinoma Mucoepidermoid, Adenoma Pleomorphic

## Abstract

**Statement of the Problem:**

Mast cells are round to elliptical cells that originate from bone marrow stem cells and enter the peripheral blood. By releasing inflammatory mediators, these cells are involved in type I hypersensitivity, wound healing, defense against pathogens, increased blood vessel formation, and destruction of the extracellular matrix. There are contradictory results regarding the role of mast cells in tumor lesions.

**Purpose:**

Considering the contradictory results and few studies on the density of mast cells in salivary tumors, the present study investigated and compared the density of mast cells in two common salivary gland tumors.

**Materials and Method:**

In the cross-sectional study after reviewing the records of patients referred to the Pathology Department of the School of Dentistry and Shahid Sadoughi Hospital in Yazd, 15 blocks of each of the mucoepidermoid carcinoma and pleomorphic adenoma tumors were taken. After Giemsa staining of the samples, the average of stained cells in 10 random fields under 400× magnification was counted. The results were analyzed using statistical tests of t-test, ANOVA, Chi-square, and Mann-Whitney in SPSS ver. 22.

**Results:**

The average mast cell counts in pleomorphic adenoma (4.2) was higher than muco-epidermoid carcinoma (1.7) but there was no significant relationship (*p*= 0.305). In mucoepidermoid carcinoma, the numbers of mast cells increased with increasing tumor grade (low: 0/467 moderate: 1/567 high: 2/983) and there was a significant relationship (*p*= 0.009).

**Conclusion:**

According to the results of the present study, it seems that the mast cells accumulation may be secondarily associated with inflammatory responses due to cell accumulation and tissue destruction by tumor cells.

## Introduction

Mast cells (MCs) are inflammatory cells originating in the bone marrow that are present in most human tissues [ 1 ]. Mastzella first discovered granule-containing cells in connective tissue in 1877 and named them Paul Ehrlich [ 2 ]. These cells are involved in inflammatory reactions and immune responses such as type 1 hypersensitivity, wound healing, and defense against pathogens [ 3 ]. Studies have shown that some mediators in MCs granules such as tryptase, kimase, vascular endothelial growth factor, fibroblast growth factor 2, and transforming growth factor β stimulate the proliferation of fibroblasts, fibrosis, and angiogenesis. Tryptase is an important growth factor for epithelial cells and can lead to the formation of new blood vessels in the tumor. Overall, large number of proteases can cause extracellular matrix degeneration and an increase in tumor size [ 1 ].

Salivary gland tumors are an important group of head and neck cancers [ 4 ] .In general, the tumor growth and metastasis is regulated when a balance is reached between protumorigenic and antitumorigenic factors. Local inflammation at the tumor site leads to the infiltration of various inflammatory cells including MCs [ 5 ]. A number of tyrosine kinase inhibitors, as anti-cancer drugs, suppress MCs by inhibiting C-Kit. Considering the possible effect of MCs on cancer cells and stroma, in recent years, the quantity of MCs in head and neck tumors such as squamous cell carcinoma and melanoma has been studied [ 1 ] .However, contradictory results have been obtained regarding the role of MCs in tumor progression [ 5 ]. Considering the contradictory results and few studies on counting and investigating the role of MCs in salivary gland tumors, the present study investigated the MCs density in two common salivary gland tumors, including mucoepidermoid carcinoma (MEC) and pleomorphic adenoma (PA).

## Materials and Method

In the present cross-sectional and analytical study, 30 samples of salivary gland tumors including 15 PAs and 15 MECs were requested from the Pathology Department of the School of Dentistry and Shahid Sadoughi Hospital in Yazd. Background information including age, sex, site of involvement, and grade of microscopic differentiation were extracted from patients' records. After preparing 4-μm sections and dehydration and paraffin removal, samples underwent 5% Giemsa staining for 30 minutes at 37°C. Two pathologists counted MCs and their average number in 10 random fields with 400× magnification. After data collection, the results were analyzed using statistical tests including t-test, ANOVA, Chi-square, and Mann-Whitney in SPSS version 22.

## Results

**Figure 1:**
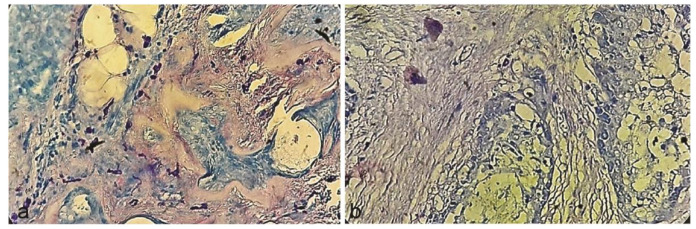
**a:** Mast cells with purple granules in pleomorphic adenoma and **b:** Mucoepidermoid carcinoma (Giemsa stain. 400×)

**Figure 2:**
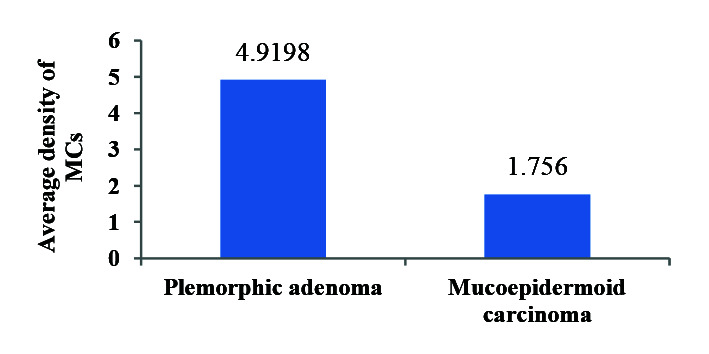
Comparison of mean mast cells (MCs) in pleomorphic adenoma and mucoepidermoid carcinoma

**Figure 3:**
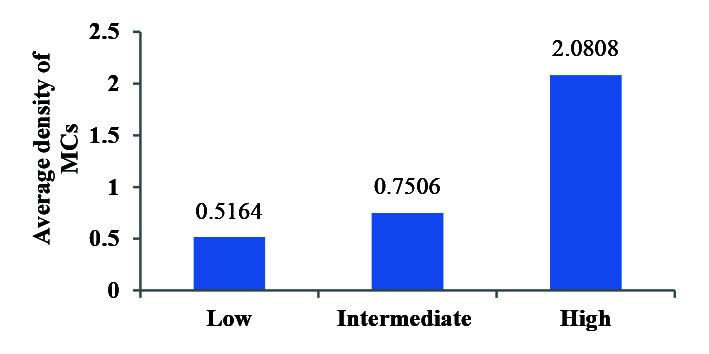
Comparison of the mean number of mast cells (MCs) in different microscopic grades of mucoepidermoid carcinoma

The present study was performed on 15 PA and 15 MEC samples. The demographic information of the samples is given in Table 1. After Giemsa staining, round, oval, or spindle-shaped MCs with purple granules were counted (Figure 1). The average MCC in PA (4.2) was higher than MEC (1.7) but no significant difference was observed between the two groups (*p*= 0.305) (Figure 2). Statistical analysis did not show a significant relationship between the average MCC and the age variable (Table 2). The average MCC in the major and minor salivary glands as well as in men and women is given in Table 3. Mann-Whitney test did not show a significant relationship between the average MCC and the sex variable. Although the average MCC was higher in the major glands, non-parametric analyzes showed that this difference was not significant. The comparison of the average MCC in different microscopic grades of MEC (low: 0/467 moderate: 1/567 high: 2/983) showed a significant difference between the three groups (*p*= 0.009) (Figure 3). 

**Table 1:** Demographic data of pleomorphic adenoma and mucoepidermoid carcinoma

Variable		Mucoepidermoid carcinoma	Pleomorphic adenoma
Group			
Age	>40	4	9
	40-70	10	6
	70-100	1	0
Sex	Female	6	8
	Male	9	7
Site of involvement	Major	11	10
	Minor	4	5
Grade of microscopic Differentiation	Low	6	
	Moderate	3	
	High	6	

**Table 2:** Relationship between mean number of mast cell and age variable

Group	Variable	Correlation coefficient	*p* Value
Pleomorphic adenoma	Age	-0.027	0.924
Mucoepidermoid		-0.175	0.534
Carcinoma			

**Table 3:** Relationship between mean number of mast cell in pleomorphic adenoma and mucoepidermoid carcinoma with sex and tumor site

Variable		Pleomorphic Adenoma (Mean±SD)	Mucoepidermoid Carcinoma (Mean±SD)	*p* Value
Sex	Female	5.62±5.15	1.63±0.98	0.199: PA
	Male	2.57± 4.4	1.73±2.18	0.550:MEC
Location	Major	5.2± 1.7	1.95±0.57	0.371:MEC
	Minor	2.2± 1.12	1.00±0.57	0.412: PA

## Discussion

The results of the present study showed that although the number of MCs in PA is higher than MEC, there is no significant relationship. In addition, the relationship between sex, age, location, and MCs density was not significant in both groups. Comparison of the average MCC in different microscopic grade of MEC showed that the average MCC increases significantly with increasing microscopic grade. 

Similar to the present study, Jaafari *et al*. [ 1 ] and Amanpour *et al*. [ 6 ] showed a higher mean number of MCs in PA than MEC, although the results of these studies were not significant [ 1 , 6 ] While Seifi *et al*. [ 5 ] and Vidal *et al*. [ 7 ] showed higher MCs density in malignant tumors than benign ones [ 5 , 7 ].

In Seifi’s *et al*. [ 5 ] and Amanpour’s *et al*. [ 6 ] study, the number of mast cells in the lower grades was more than the high grades, which is consistent with the present study and Yazdani *et al*. [ 5 - 6 , 8 ]. Contradictory results between different studies can be due to differences in sample size, tissue preparation methods including fixation, antigen recycling, staining technique, and the type of staining used. In the present study, Giemsa staining was used, while in other studies, other types of staining were used, including toluene blue, Alcian blue, azure, and so on [ 9 - 12 ]. In a number of other studies, immunohistochemistry technique has been used [ 5 , 13 - 17 ] .In the present study, like the Jafari *et al*. [ 1 ], MCs were counted in 10 random fields, while in the studies of Seifi *et al*. [ 5 ] and Amanpour *et al*. [ 6 ], MCs counting were performed in four random fields [ 1 , 5 - 6 ]. 

In a study of normal mucosa, mild to severe dysplasia, and squamous cell carcinoma, Gomes *et al*. [ 18 ] found that MCs density was higher in oral squamous cell carcinoma than in other groups. These researchers believed that the presence of MCs was important for tumor growth and invasion, but it is not related to grades of tumor differentiation [ 18 ].

Mohtasham *et al*. [ 19 ] concluded that the MCs density in high microscopic grades is more than low grades of oral squamous cell carcinoma and this cell can play a role in the formation and progression of tumors through angiogenesis [ 19 ].

Nike *et al*. [ 20 ] also reported an inverse relationship between MCs density and the malignancy grade of tumors, including squamous cell carcinoma of the cervix. They suggested that these cells might be related to cell differentiation [ 20 ]. However, Sharma *et al*.[ 21 ] found a direct relation between MC density and microscopic grade of oral squamous cell carcinoma[ 21 ]. 

Regarding the role of MCs in the formation of malignant tumors, Ranieri *et al*. [ 11 ] and Coussens *et al*. [ 12 ] believe that MCs infiltration in the early stages of tumor formation is due to immune system stimulation and cytotoxic properties of these cells; however, those researchers believe that MCs density decreases with increasing malignancy grade. They attribute this reduction to the fact that long-term exposure of the MC to the tumor antigen reduces its sensitivity and creates a kind of adaptation in this cell, and gradually reduces the migration of this cell around the tumor. Another cause of the average MCC reduction includes the degranulation and impairment in their diagnosis and counting [ 11 - 12 ]. MCs-secreted mediators such as vascular endothelial growth factor and interleukin 8 can play a role in the formation of blood vessels and suppression of the immune system and provide the conditions for tumor formation [ 12 ]. On the other hand, all the above studies are descriptive-cross-sectional and cannot prove any cause-effect relationship between the presence of MCs and the differentiation grade of malignant tumors or the pathogenesis of benign and malignant salivary tumors. The MC accumulation may be a secondary phenomenon due to the stimulation of the immune system. Despite the fact that the number MCs is higher in PA than MEC in the present study, some studies [ 23 - 25 ] have shown that the angiogenesis rate in MEC is higher than in PA, there is enough evidence to prove the role of MCs angiogenesis, increased malignancy and decreased tumor differentiation.

It is reported that there are high numbers of MCs in PAs with a variable stroma. The lack of significant differences between the two study groups is probably due to variable stroma in PA, which leads to an increase in the average number of MCs [ 1 ].

The exact role of MCs in tumor development and growth is critical to new goals for cancer treatment. Although MCs can be a viable target for new therapies, there is still limited information on the treatment of human cancers. From a therapeutic point of view, the use of MC inhibitors such as tyrosine kinase inhibitors (imatinib and masitinib) to inhibit the C-Kite receptor and MC tryptase inhibitor (Tran last) may reduce tumor growth, angiogenesis, and metastasis [ 26 - 31 ].

## Conclusion

Considering to higher number of MCs in benign tumors than malignant ones and increase of cells with increasing degree of malignancy, it seems that the MCs accumulation is related to the inflammatory responses caused by cell accumulation and tissue destruction by tumor cells and it has slight relationship with tumor growth and development.

## Conflict of Interest

Authors declare that there are no conflicts of interest in this study. 

## References

[1] Jaafari-Ashkavandi Z, Ashraf MJ ( 2014). Increased mast cell counts in benign and malignant salivary gland tumors. J Dent Res Dent Clin Dent Pros.

[2] Mahita V, Manjunatha B, Shah R, Astekar M, Purohit S, Kovvuru S ( 2015). Quantification and localization of mast cells in periapical lesions. Ann Med Health Sci Res.

[3] Kouhsoltani M, Khiavi MM, Jamali G, Farnia S ( 2015). Immunohistochemical assessment of mast cells and small blood vessels in dentigerous cyst, odontogenic keratocyst, and periapical cyst. Adv Pharm Bull.

[4] Ismerim AB, de Oliveira Araújo IB, de Aquino Xavier FC, Rocha CA, Macedo CL, Cangussu MC, et al ( 2021). Mast Cells and Proteins Related to Myofibroblast Differentiation (PAR-2, IL-6, and TGFβ1) in Salivary Cancers: A Preliminary Study. Appl Immunohistochem Mol Morphol.

[5] Seifi S, Yazdani F, Bijani A, Rayat N, Amini Shakib P ( 2013). Comparison and evaluation of mast cell density in pleomorphic adenoma, mucoepidermoid carcinoma and adenoid cystic carcinoma of salivary gland with toluidine blue staining. J Babol Univ Med Sci.

[6] Amanpour S, Rad M, Mirshekari TR, Torabi-Parizi M, Fardisi S, Shafazand S ( 2017). Survey of Mast Cells Density in the Most Prevalent Benign and Malignant Salivary Gland Tumors in Southeast of Iran, 2005-2015. J Rafsanjan Univ Med Sci.

[7] Vidal MT, de Oliveira Araújo IB, Gurgel CA, Pereira Fde A, Vilas-Bôas DS, Ramos EA, et al ( 2013). Density of mast cells and microvessels in minor salivary gland tumors. Tumour Biol.

[8] Yazdani F, Rezvani G, Safari S, Jaberi M ( 2015). Histopathological comparision of mast cell density in various grades of mucoepidermoid carcinoma. Cancer Res J.

[9] Katopodi E, Kavantzas N, Pavlopoulos P, Papanikolaou V, Saetta A, Korkolopoulou P, et al ( 2004). The frequency and distribution of mast cells in pleomorphic adenomas of salivary glands. Pathology.

[10] Ashkavandi Z, Jaafari-Moshref M, Mashhadi-Abbas F, Sargolzaie S, Taghavi N ( 2010). Evaluation of CD31 expression and mast cell count in dysplastic lesions and squamous cell carcinoma of the oral cavity. Iranian Red Crescent Med J.

[11] Ranieri G, Labriola A, Achille G, Florio G, Zito AF, Grammatica L, et al ( 2002). Microvessel density, mast cell density and thymidine phosphorylase expression in oral squamous carcinoma. Int J Onco.

[12] Coussens LM, Raymond WW, Bergers G, Laig-Webster M, Behrendtsen O, Werb Z, et al ( 1999). Inflammatory mast cells up-regulate angiogenesis during squamous epithelial carcinogenesis. Genes Dev.

[13] Kabiraj A, Jaiswal R, Singh A, Gupta J, Singh A, Samadi FM ( 2018). Immunohistochemical evaluation of tumor angiogenesis and the role of mast cells in oral squamous cell carcinoma. J Cancer Res Ther.

[14] Fukushima H, Ohsawa M, Ikura Y, Naruko T, Sugama Y, Suekane T, et al ( 2006). Mast cells in diffuse large B‐cell lymphoma; their role in fibrosis. Histopathology.

[15] Ribatti D, Ennas MG, Vacca A, Ferreli F, Nico B, Orru S, et al ( 2003). Tumor vascularity and tryptase‐positive mast cells correlate with a poor prognosis in melanoma. Eur J Clin Invest.

[16] Nonomura N, Takayama H, Nishimura K, Oka D, Nakai Y, Shiba M, et al ( 2007). Decreased number of mast cells infiltrating into needle biopsy specimens leads to a better prognosis of prostate cancer. British J Cancer.

[17] Rajput AB, Turbin DA, Cheang MC, Voduc DK, Leung S, Gelmon KA, et al ( 2008). Stromal mast cells in invasive breast cancer are a marker of favourable prognosis: a study of 4,444 cases. Breast Cancer Res Treat.

[18] Gomes AP, Johann JE, Lovato GG, Ferreira AM ( 2008). Comparative analysis of the mast cell density in normal oral mucosa, actinic cheilitis and lip squamous cell carcinoma. Brazilian Dent J.

[19] Mohtasham N, Babakoohi S, Salehinejad J, Montaser-Kouhsari L, Shakeri MT, Shojaee S, et al ( 2010). Mast cell density and angiogenesis in oral dysplastic epithelium and low- and high-grade oral squamous cell carcinoma. Acta Odontol Scand.

[20] Naik R, Pai MR, Poornima Baliga B, Nayak KS, Shankarnarayana, Dighe P ( 2004). Mast cell profile in uterine cervix. Indian J Patho Micro.

[21] Sharma B, Sriram G, Saraswathi TR, Sivapathasundharam B ( 2010). Immunohistochemical evaluation of mast cells and angiogenesis in oral squamous cell carcinoma. Indian J Dent Res.

[22] Theoharides TC, Conti P ( 2004). Mast cells: the Jekyll and Hyde of tumor growth. Trends Immunol.

[23] Tadbir AA, Pardis S, Ashkavandi ZJ, Najvani AD, Ashraf MJ, Taheri A, et al ( 2012). Expression of Ki67 and CD105 as proliferation and angiogenesis markers in salivary gland tumors. Asian Pac J Cancer Prev.

[24] Moghadam SA, Abadi AM, Mokhtari S ( 2015). Immunohistochemical analysis of CD34 expression in salivary gland tumors. J Oral Maxillofac Patho (JOMFP).

[25] Ota T, Ota K, Jono H, Fujimori H, Ueda M, Shinriki S, et al ( 2010). Midkine expression in malignant salivary gland tumors and its role in tumor angiogenesis. Oral Oncology.

[26] Ammendola M, Sacco R, Sammarco G, Luposella M, Patruno R, Gadaleta CD, et al ( 2016). Mast cell-targeted strategies in cancer therapy. Transfus Med Hemother.

[27] Patruno R, Marech I, Zizzo N, Ammendola M, Nardulli P, Gadaleta C, et al ( 2014). c-Kit expression, angiogenesis, and grading in canine mast cell tumour: a unique model to study c-Kit driven human malignancies. BioMed Res Int.

[28] Josephs DH, Fisher DS, Spicer J, Flanagan RJ ( 2013). Clinical pharmacokinetics of tyrosine kinase inhibitors: implications for therapeutic drug monitoring. Ther Drug Monit.

[29] Marech I, Gadaleta CD, Ranieri G ( 2014). Possible prognostic and therapeutic significance of c-Kit expression, mast cell count and microvessel density in renal cell carcinoma. Inte J Molecular Sci.

[30] Arock M, Sotlar K, Akin C, Broesby-Olsen S, Hoermann G, Escribano L, et al ( 2015). KIT mutation analysis in mast cell neoplasms: recommendations of the European Competence Network on Mastocytosis. Leukemia.

[31] Ammendola M, Leporini C, Marech I, Gadaleta CD, Scognamillo G, Sacco R, et al ( 2014). Targeting mast cells tryptase in tumor microenvironment: a potential antiangiogenetic strategy. BioMed Res Int.

